# Regulation Mechanisms of Plant Basic Leucine Zippers to Various Abiotic Stresses 

**DOI:** 10.3389/fpls.2020.01258

**Published:** 2020-08-20

**Authors:** Yan Yu, Yuchen Qian, Mengyue Jiang, Jia Xu, Jingting Yang, Tongyao Zhang, Liangpeng Gou, Erxu Pi

**Affiliations:** College of Life and Environmental Sciences, Hangzhou Normal University, Hangzhou, China

**Keywords:** plant, basic leucine zipper transcription factor, *cis*-element, stress tolerance, flavonoid

## Introduction

In the process of growth and development, plants are exposed to various abiotic stresses such as salinity, drought, low temperature, which limit crop yield and quality. During evolution, plants acquire series of resistances to these environmental stresses and survive through physiological, biochemical, and molecular responses. These responses are usually originated by regulating the expression of relevant genes. bZIP (basic leucine zipper) transcription factors, as one of the largest transcription factor regulatory families, play very important roles in responses to these abiotic stresses. bZIP TFs could be activated by drought, high salt and chilling damages. By binding specifically to *cis*-elements in the promoter region of stress related genes, they can regulate the transcriptional expressions of target genes, thereby regulating stress resistance of plants. This article comprehensively reviews the structural characteristics of bZIPs and their regulation mechanisms on target genes under various abiotic stresses.

## Distribution and Classification of bZIP Transcription Factors

Currently, there are at least 64 families of transcription factors have been found in plants (Pérez-Rodriguez et al., 2010). According to their differences in DNA-binding domains, transcription factors can be defined as different families, such as bZIP, NAC, MYB, EREBP/AP2, Zinc-finger, etc. To date, a large number of bZIP transcription factors have been identified in almost all eukaryotes. There are 57, 77, 62, 96, 85, 87, 89, 262, 92, 89, 178, 103, 65, 69, 125, 64, 55, 114 bZIP transcription factors been found in *Ananas comosus*, *Arabidopsis thaliana*, *Citrullus lanatus*, *Fagopyum talaricum, Gossypium raimondii, Gossypium arboreum, Oryza sativa*, *Glycine max*, *Sorghum bicolor*, *Hordeum vulgare* L, *Panicum virgatum* L, *Olea europaea* L, *Solanum tuberosum* L., *Solanum lycopersicum*, *Zea mays*, *Cucumis sativus*, *Vitis vinifera* and *Malus domestica*, respectively (Corrêa et al., 2008; Nijhawan et al., 2008; Wang et al., 2011; Wei et al., 2012; Baloglu et al., 2014; Liu J. Y. et al., 2014; Li et al., 2015; Pourabed et al., 2015; Li et al., 2016; Zhang et al., 2018; Liu M. et al., 2019; Yang W. et al., 2019; Azeem et al., 2020; Liu et al., 2020; Rong et al., 2020; Wang et al., 2020; Zhao et al., 2020). Only 25, 21, and 21 bZIP transcription factors were found in yeast, nematode, and fruit fly, respectively (Riechmann et al., 2000). Compared to other eukaryotes, plants seem to have more bZIP homologous proteins and more conserved amino acid sequences in these homologies (Ali et al., 2016). Studies have shown that the structures of bZIP protein are closely related to its biological function. Jakoby et al. (2002) used MEME (multiple em for motif elicitation) to analyze a large number of bZIP transcription factors in *Arabidopsis thaliana*. Based on the characteristics of both the bZIP and other conserved motifs, the 75 bZIPs in *Arabidopsis thaliana* were classified into 10 subfamilies (A, B, C, D, E, F, G, H, I, and S). With similar method, the bZIP transcription factor family genes in other plants have also been categorized. The 131 bZIP transcription factors isolated from the soybean genome were also divided into abovementioned 10 subfamilies A~S (Liao et al., 2008). Though the 89 members of the bZIP transcription factor family in rice were also divided into 10 subfamilies, the subfamily S was replaced with J (Nijhawan et al., 2008). It seems that most of these subfamilies of bZIPs are conserved among different plants. Corrêa et al. (2008) identified the possible non-redundant complete sets of 92 bZIPs in rice and 89 bZIPs in black cottonwood. Based on the similarities of both bZIP and other conserved motifs, these collections of bZIPs together with the 77 bZIPs from Arabidopsis were categorized into 13 subfamilies, including A, B, C, D, E, F, G, H, I, J, K, L, and S. In which, three subgroups including J, K, and L were added.

With the advancement of bioinformatics, more and more conversed motifs, except bZIP, were identified for categorizing bZIP subfamilies. Hence, the classification of bZIP transcription factors has become more and more sophisticated. Due to the advancement of bioinformatics, there are increasing researches provide preliminary analyses on globally identifying bZIP members from the fresh released genomic database of many plants, such as potato, switchgrass, olive, pineapple, cotton, watermelon, and tartaty buckwheat, laying the foundation for subsequent research (Yang W. et al., 2019; Liu M. et al., 2019; Azeem et al., 2020; Liu et al., 2020; Rong et al., 2020; Wang et al., 2020; Zhao et al., 2020). Recent years, there are increasing reports on regulation mechanism of various bZIPs on different stress responses (Liu et al., 2012; Ji et al., 2013; Hwang et al., 2014; van Leene et al., 2016; Tsugama et al., 2016; Zhang C. Y. et al., 2017; Zhang L. N. et al., 2017; Wang et al., 2019). Specific roles of bZIPs in different subgroups might also be categorized into corresponding biological pathways, considering plenty of functional annotated bZIPs been classified into the known subfamilies with those sophisticated bioinformatics.

## Architecture Characteristics of bZIP Transcription Factors

Transcription factor, also known as *trans*-acting factor, is a category of proteins that can specifically bind to *cis*-acting elements in the promoter region of eukaryotic genes, thereby activating or silencing the expression of related genes with temporal and spatial specificity. The structure of plant transcription factors generally includes at least four functional domains, including the DNA binding domain, the transcriptional regulatory domain, the nuclear localization signal peptide, and the oligomerization site (Du et al., 2012). They work together to regulate various biological processes.

Although the classification of bZIPs varies depending on the researcher’s choice of criterions, there is currently a consensus on this family that their protein sequence contains a conserved bZIP domain with 60~80 amino acids length. This domain is consisted of at least two specific structures. Firstly, the N-terminus is a basic region composed of about 20 basic amino acids, containing a nuclear localization signal (NLS) and a N-x7-R/K structural unit that specifically binds to a DNA sequence. This region is involved in nuclear localization and DNA binding (Lee S. C. et al., 2006). Secondly, the C-terminus, which is a leucine zipper region, a heptad repeat of leucine or other bulky hydrophobic amino acids (Ile, Val, Phe, or Met), creates an amphipathic helix. This region is involved in the dimerization of the bZIP protein before it binds to DNA (Landschulz et al., 1988; Hurst, 1994; Jakoby et al., 2002). In addition to the bZIP domain, the bZIPs also contain other conserved domains with transcriptional activation functions, such as the R/KxxS/T and S/TxxD domains, which are phosphorylation sites of Ca^2+^ independent protein kinase and casein kinase II (Furihata et al., 2006). Besides, there are also some regions rich in acidic amino acids, which can activate the transcriptional expression of downstream target genes (Liao et al., 2008).

## Mechanisms of bZIP on Transcriptional Regulation of Target Genes

Through dimerization, phosphorylation, or interaction with other nuclear proteins, the specificity and affinity of bZIP binding to DNA will change, which will affect the activation of other genes, as well as its own stability and subcellular localization (Schütze et al., 2008). By forming homo- or heterodimers and binding specific promoters in its basic region, the bZIP transcription factor inhibits or activates the expression of target genes.

The binding specificity of bZIP factors in plants is mainly determined by three bases flanking the four core nucleotides. Generally, bZIP factors preferentially select ACGT core palindromes or pseudo-palindromic *cis*-acting elements to bind, such as G-box (CACGTG), C-box (GACGTC), A-box (TACGTA), ABRE (ACGTGGC) (Izawa et al., 1993; Kim et al., 2004). Most of them are located in the ABA hormone-induced promoter region. When the bZIP protein interacts with these *cis*-acting elements, the N-terminus of its basic domain is inserted into the large groove of the DNA double-strand, and the C-terminus of the leucine zipper is dimerized to form a superimposed curl helix (Landschulz et al., 1988; Ellenberger et al., 1992).

G-box is one of the most common targets of bZIP transcription factors. de Vetten and Ferl (1995) firstly found that corn GBF1 is a basic region leucine zipper protein and could activate *Adhl* expression by binding to its G-box. After that, series of stress related genes were found to be bound at their G-box and regulated by various bZIPs. Kaminaka et al. (2006) found that *Arabidopsis thaliana AtbZIP10* can combine with G-box to negatively regulate plant resistance to pathogenic bacteria and other stresses. Zou et al. (2008) demonstrated that the rice OsbZIP10/OsABI5 could bind to the G-box element for *trans*-activating stress resistance genes, thereby inhibiting seed germination and seedling growth. Liu et al. (2012) also found that *OsbZIP52*/*RISBZ5* can recognize the G-box on target genes to enhance the low temperature sensitivity of rice. The *Arabidopsis thaliana* AtbZIP56/HY5 binds directly to the promoters of light responsible element containing the G-box and thus regulates their transcriptional activity (Yoon et al., 2006). Induced by salt, the *Tamarix hispida* bZIP1 bound to G-box of the stress response genes and regulated their expression (Ji et al., 2013). Using chromatin immunoprecipitation, Lee et al. (2006a) demonstrated that CabZIP1 bound to the G-box elements in native promoter of the hot pepper *pathogenesis-related protein* 1 (*CaPR-1*) gene *in vivo*. Shaikhali et al. (2012) identified the AtbZIP16 as a component binding to the G-box-containing promoter fragment of light-harvesting chlorophyll a/b-binding protein2.4 (LHCB2.4) from nuclear extracts of high light-treated Arabidopsis plants.

The ABRE element is also a favorite target of bZIP transcription factors. Sun et al. (2011) found that AtbZIP1 binds to ABRE active elements and regulates the plant’s response to low temperature stress through ABA-dependent signaling pathways. Yoshida et al. (2015) demonstrated that the *Arabidopsis thaliana* bZIP transcription factors ABF1, ABF2, ABF3, and ABF4 combined with ABRE and regulated the expression of downstream genes related to salt and drought tolerance. In maize, *ZmbZIP17* functions as an ER stress transducer, interacting with ABREs (Yang et al., 2013). Rice OsbZIP46/OsABF2 (Hossain et al., 2010; Tang N. et al., 2012; Chang et al., 2017), *OsbZIP52/RISBZ5* (Liu et al., 2012), *OsbZIP10/OsABI5* (Zou et al., 2007; Zou et al., 2008; Chang et al., 2019), OsbZIP05/OSBZ8 (Nakagawa et al., 1996; Mukherjee et al., 2006) could all regulate the expression of plant ABA-responsive genes by binding to their ABRE element. Zhang et al. (2017b) proved that wheat TabZIP14-B showed transcriptional activation ability through the transactivation assay and was capable of binding the ABRE in yeast. Zhang et al. (2020) found that, TabZIP8, 9, 13 could combine to the ABREs of *TaNCED2* gene to promote ABA biosynthesis in wheat roots in response to salt stress. Wang et al. (2019) isolated the sweet potato bZIP transcription factor *IbABF4* gene, and found its *cis*-acting activity on ABRE *in vitro*. Liu et al. (2019b) found that the Cassava MeABL5 was able to specifically interact with the ABRE *cis*-element in the promoter of the major cell wall invertase gene *MeCWINV3*.

In addition, bZIP transcription factors could target on genes by C-Box and A-box. The C-box of pathogenic responsive genes could bound and negatively regulated by *AtbZIP10* in *Arabidopsis thaliana* (Kaminaka et al., 2006). Induced by ABA and drought, the *Tamarix hispida* bZIP1 bound to C-box and A-box *cis*-elements of the stress response gene (Ji et al., 2013).

**Figure 1:**
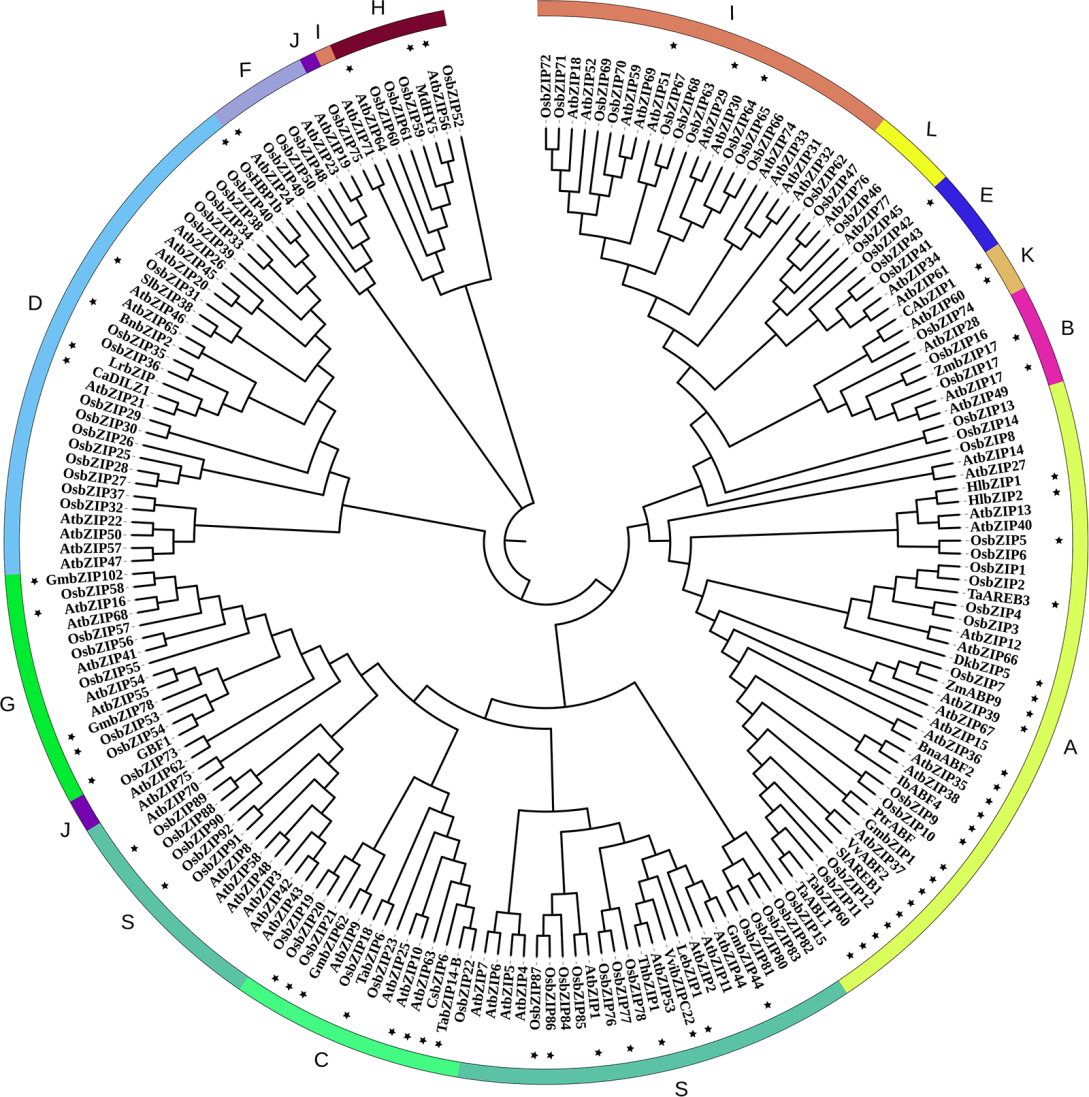
Phylogenetic relationships of bZIP family members. Concatenated sequence of all conserved motifs were used for multiple sequence alignments with the clustalX software. The phylogenetic tree was constructed using MEGA7.0 with the neighbor-joining method and 1000 bootstrap replicates (Saitou and Nei, 1987; Kumar et al., 2016). Then the trees were visualized using iTOL (https://itol.embl.de/). Group names were marked outside the circle. The bZIP protein sequences were downloaded from the JGI (http://www.jgi.doe.gov/) and NCBI (https://www.ncbi.nlm.nih.gov) databases. The Gene and Protein IDs of all these bZIPs are list in **Supplemental Tables S1****–****S3**. In this phylogenetic tree, the nomenclature of the *Oryza sativa* bZIPs is consistent with Corrêa et al., 2008 if there is any conflict with that in other publications (**Supplemental Table S1**). And the bZIP members that have been functional annotated are labeled with a star marker.

In summary, bZIP transcription factors regulate the transcriptional expression by interacting with specific *cis*-regulatory sequences in the promoter region of response genes to regulate plant stress tolerance (Sornaraj et al., 2016). To understand the actual relationship between bZIP subfamilies and their binding *cis*-regulator motifs (**Table 1** and **Figure 1**), all the functional annotated bZIPs were categorized into 13 known subgroups based on the method described by Corrêa et al. (2008). It seems that the G-Box and ABRE attracts most scientists’ interests and are two most understood *cis*-elements of bZIP transcription factors (**Table 1**). The bZIPs that bind to G-Box are most categorized into subfamilies A, C, G, H, K, and S; while those recognize ABRE usually belong to the subgroups A, B, C, G, and S (**Table 1**). Besides, there are also several reports on mechanisms about how bZIP transcription factors regulate other two *cis*-elements, C-box and A-box (**Table 1**). Interestingly, bZIPs that bind to C-box are usually belong to subfamilies C and S; the functional annotated bZIP bind to A-box is classified into subfamily S. Though the number of functional annotated bZIP is limit, their binding activities of different subfamilies to specific *cis*-elements could also provide directional suggestions for further research on *de novo* bZIPs and potential targets. However, more evidences are still needed to fulfill the relevance between bZIP subfamilies and corresponding *cis*-elements.

**Table 1:** Mechanism of bZIP on transcriptional regulation of target genes.

*cis-*acting element	Plant species	Nomenclature	Subfamily	Effect	Reference
G-box	*Zea* *mays*	GBF1	G	Activate Adhl expression	de Vetten and Ferl, 1995
G-box	*Oryza* *sativa*	OsbZIP52	C	Negatively regulated cold tolerance	Liu et al., 2012
G-box	*Oryza* *sativa*	OsbZIP10/ OSABI5	A	Inhibiting seed germination and seedling growth; Negatively regulated salt tolerance	Zou et al., 2007; Zou et al., 2008
G-box, ABRE	*Oryza* *sativa*	OsbZIP62	A	Positively regulates the rice drought and oxidative stress responses.	Yang S. et al., 2019
G-box, C-box	*Arabidopsis* *thaliana*	AtbZIP10	C	Negatively regulate plant resistance to pathogenic bacteria and other stresses	Kaminaka et al., 2006
G-box	*Arabidopsis* *thaliana*	AtbZIP56/ HY5	H	Interact with the COP1 protein for proteasome-mediated degradation in the nucleus.	Yoon et al., 2006
G-box	*Arabidopsis* *thaliana*	AtbZIP16	G	Involved in the light- and/or redox-triggered regulation of LHCB2.4 expression	Shaikhali et al., 2012
C-box, G-box, A-box	*Tamarix* *hispida*	ThbZIP1	S	Improve salt tolerance of plant	Ji et al., 2013
G-box	*Capsicum* *annuum*	CabZIP1	K	Enhanced resistance to pathogen infection and environmental stresses	Lee B. J. et al., 2006
ABRE	*Arabidopsis* *thaliana*	AtbZIP35/AtABF1, AtbZIP36/AtABF2/AREB1, AtbZIP37/AtABF3, AtbZIP38/AtABF4/AREB2	A	Involved in ABA response and stress response	Choi et al., 2000
ABRE	*Arabidopsis* *thaliana*	AtbZIP39/ AtABI5	A	Responds to ABA, drought, and salt stress	Nakashima et al., 2009
ABRE	*Arabidopsis* *thaliana*	AtbZIP1	S	Regulates the plant’s response to low temperature stress	Sun et al., 2011
ABRE	*Zea* *mays*	ZmbZIP17	B	ER stress transducer	Yang et al., 2013
ABRE	*Oryza* *sativa*	OsbZIP46/OsABF2, OsbZIP52/RISBZ5, OsbZIP05/OSBZ8	A, C, G	Involved in ABA response and stress response	Hossain et al., 2010; Chang et al., 2017; Mukherjee et al., 2006; Liu et al., 2012; Zou et al., 2007; Tang N. et al., 2012; Zou et al., 2008;
ABRE	*Triticum* *aestivum*	TabZIP14-B	C	Involved in stress response	Zhang L. N. et al., 2017
ABRE	*Triticum* *aestivum*	TabZIP8, TabZIP9, TabZIP13	A	Involved in ABA response and stress response	Zhang et al., 2020
ABRE	*Ipomoea* *batatas*	IbABF4	A	Involved in stress response	Wang et al., 2019
ABRE	*Tartary Buckwheat*	FtbZIP5	A	Enhance salt and drought tolerance	Li et al., 2020

## Regulation Mechanism of Plant bZIPS to Various Stresses

Previous studies have found that bZIPs play important roles in response to a variety of plant stresses, such as salinity, drought, and cold damages (**Table 2**). Their regulation mechanism varies depending on species of plant and types of stresses.

**Table 2:** bZIP transcription factors involved in plant abiotic stress response.

Original Plant	Stress response	Nomenclature	Subfamily	Target gene	Regulation type	Function	Reference
*Arabidopsis thaliana*	Salt	AtbZIP17	B	*ATHB-7*	Positive regulation	Improve salt tolerance of plant	Liu et al., 2007; Liu et al., 2008
*Arabidopsis thaliana*	Salt	AtbZIP24	F	Unknown	Negative regulation	Participate in salt stress response	Yang et al., 2009
*Arabidopsis thaliana*	Drought	AtbZIP37/ AtABF3	A	*ABI 5*	Positive regulation	Enhance drought tolerance	Wang Z. et al., 2016 Chang et al., 2019
*Arabidopsis thaliana*	Salt	AtbZIP60	K	Unknown	Positive regulation	Enhance salt tolerance	Tang W. et al., 2012
*Arabidopsis thaliana*	Salt	AtbZIP62	J	GLS 1, S*OS1*, *SOS2*, *SOS3*,	Negative regulation	Participate in salt stress response	Rolly et al., 2020
*Arabidopsis thaliana*	Osmotic stress	AtbZIP63	C	Unknown	Positive regulation	Enhance osmotic tolerance	Veerabagu et al., 2014
*Arabidopsis thaliana*	Osmotic stress	AtbZIP51/ VIP1, AtbZIP29	I	*CYP707A1, CYP707A3*	Unknown	Participate in osmotic stress response	Hwang et al., 2014; Van Leene et al., 2016; Tsugama et al., 2016
*Arabidopsis thaliana*	Cold	AtbZIP1	S	Unknown	Negative regulation	Participate in cold stress response	Sun et al., 2011
*Arabidopsis thaliana*	mechanical stress	VIP 1	I	Unknown	Negative regulation	suppresses mechanical stress-induced root waving	Tsugama et al., 2019
*Boehmeria nivea*	Salt Drought	BnbZIP2	D	Unknown	Positive Regulation (salt) Negative regulation (drought)	Participate in salt and drought stress response	Huang et al., 2016
*Brassica napus*	Salt	BnaABF2	A	Unknown	Positive regulation	Enhance salt tolerance	Zhao et al., 2016
*Brassica rapa*	Cold	Bra000256	I	Unknown	Unknown	Participate in cold stress response	Hwang et al., 2014
*Camellia sinensis*	Cold	CsbZIP6	C	Unknown	Negative regulation	Participate in cold stress response	Wang L. et al., 2017
*Camellia sinensis*	Salt/ Drought/ Cold	CsbZIP18	K	*AAO3*, *CYP707A3*, *UGT71B6, ABCG 22*	Negative regulation	Participate in cold stress response	Yao et al., 2020
*Capsicum annuum*	Drought	CaDILZ1	D	Unknown	Negative regulation	Participate in drought stress response	Lim et al., 2018
*Capsicum annuum*	Salt/ Drought	CAbZIP1	K	Unknown	Positive regulation	Enhance salt and drought tolerance	Lee S. C. et al., 2006
*Capsicum annuum*	Salt	CabZIP 25	A	Unknown	Positive regulation	Enhance salt tolerance	Gai et al., 2020
*Glycine max*	Salt/Cold	GmbZIP44, GmbZIP62, GmbZIP78	S,C,G	*ABI1, ABI2*	Positive regulation	Enhance salt resistance	Wang et al., 2015
*Glycine max*	Salt/ Drought/Cold	GmbZIP1	A	Unknown	Positive regulation	Enhance salt, drought cold resistance	Gao et al., 2011
*Glycine max*	Drought	GmbZIP102	G	Unknown	Positive regulation	Participate in drought response	Zhang et al., 2018
*Glycine max*	Salt/ Drought/ Cold/ ABA	GmbZIP2	G	GmMYB48, GmWD40, GmDHN15, GmGST1 GmLEA	Positive regulation	Enhance salt, drought resistance.	Yang et al., 2020
*Nymphaea nelumbo*	Salt	LrbZIP	D	Unknown	Positive regulation	Enhance salt tolerance	Cheng et al., 2013
*Malus pumila*	Cold	MdHY5	H	*MdCBF1, CORs*	Positive regulation	Enhance cold tolerance	An et al., 2017b
*Manihot esculenta Crantz*	Drought/ ABA	MeABL 5	A	*MeCWINV 3*	Positive regulation	Participate in abiotic stresses.	Liu J. et al., 2019
*Oryza sativa*	Salt	OsbZIP05/ OSBZ8	G	Unknown	Positive regulation	Rapidly induced by abscisic acid; salt tolerance	Nakagawa et al., 1996; Mukherjee et al., 2006
*Oryza sativa*	Salt/ Drought	OsbZIP71	S	*OsNHX1, COR413-TM1*	Positive regulation	Enhance salt and drought tolerance	Liu C. T. et al., 2014
*Oryza sativa*	Salt	OsHBP1b	D	Unknown	Positive regulation	Enhance salt tolerance	Lakra et al., 2015
*Oryza sativa*	Salt/ Drought/ABA	OsbZIP16	S	Unknown	Positive regulation	Participate in salt and drought stress response	Chen et al., 2012; Pandey et al., 2018
*Oryza sativa*	Cold	OsbZIP38/ OsLIP19	S	Unknown	Positive regulation	involved in cold signaling; a fos-like molecular switch in the cold signaling	Aguan et al., 1991; Aguan et al., 1993; Shimizu et al., 2005
*Oryza sativa*	Cold	OsbZIP87/ OsOBF1	S	Unknown	Negative regulation	interact with lip19 and involved in cold signaling	Shimizu et al., 2005
*Oryza sativa*	Cold/ Drought	OsbZIP52/ RISBZ5	C	Unknown	Negative regulation	Participate in cold and drought stress response	Liu et al., 2012
*Oryza sativa*	Cold	OsbZIP68/ ROS-bZIP1	I	Unknown	Positive regulation	induced by low temperature and hydrogen peroxide in seedlings of chilling-tolerant japonica rice	Cheng et al., 2007
*Oryza sativa*	Drought	OsbZIP72	A	Unknown	Positive regulation	Positive regulator of ABA response and drought tolerance in rice	Lu et al., 2009
*Oryza sativa*	Cold	OsbZIP73/ OsTFX1	S	Unknown	Positive regulation	Enhance cold resistance	Liu et al., 2018; Liu C. T. et al., 2019
*Oryza sativa*	Salt	OsbZIP12/ OsABF1	A	*COR413-TM1*	Positive regulation	Inhibit rice flowering; enhance salt resistance	Amir Hossain et al., 2010; Zhang C. Y. et al., 2017
*Oryza sativa*	Drought	OsbZIP20	C	Unknown	Unknown	Participate in drought stress response	Izawa et al., 1993
*Oryza sativa*	Drought/ Salt	OsbZIP23	A	*OsPP2C49 etc*.	Positive regulation	Enhance salt and cold tolerance	Xiang et al., 2008; Dey et al., 2016; Zong et al., 2016
*Oryza sativa*	Drought	OsbZIP46/ OsABF2/ ABL1	A	Unknown	Positive regulation	Co-overexpression with SAPK6 to enhance drought tolerance	Hossain et al., 2010; Tang N. et al., 2012; Chang et al., 2017
*Oryza sativa*	Drought	OsbZIP42	E	Unknown	Positive regulation	Enhance drought tolerance	Joo J. S. et al., 2019
*Oryza sativa*	Salt/ Drought/ABA	OsbZIP10/ OsABI5	A	Unknown	Negative regulation	Participate in salt and drought stress response	Zou et al., 2007; Zou et al., 2008
*Oryza sativa*	Salt	OsbZIP62	A	DSM 2, *OsNAC 10*, *OsGL 1*	Positive regulation	Enhanced drought tolerance	Yang S. et al., 2019
*Poncirus trifoliata*	Drought	PtrABF	A	Unknown	Positive regulation	Enhance drought resistance	Huang et al., 2010
*Solanum lycopersicum*	Salt/ Drought	SlAREB1	A	Unknown	Positive regulation	Participate in salt and drought stress response	Hsieh et al., 2010
*Solanum lycopersicum*	Salt/ drought	SlbZIP38	D	Unknown	Negative regulation	Participate in salt and drought stress response	Pan et al., 2017
*Solanum lycopersicum*	Cold	LebZIP1	S	Unknown	Unknown	Participate in cold stress response	Stanković et al., 2000
*Ipomoea batatas*	Drought	IbABF4	A	Unknown	Positive regulation	Enhance stress tolerance	Wang et al., 2019
*Ipomoea batatas*	Drought/ Salt	IbbZIP 1	E	*NCED, ABA2*, *P5CS, SOD*, *GPX, CAT*, *APX, DHAR*	Positive regulation	Enhance salt, drought resistance	Kang et al., 2019
*Tamarix hispida*	Salt/ Drought	ThbZIP1	S	Unknown	Positive regulation	Enhance salt and drought tolerance	Wang et al., 2010; Ji et al., 2013
*Triticum aestivum*	Salt	TabZIP8, TabZIP9, TabZIP13	A	*TaNCED2*	Positive regulation	Enhance salt tolerance	Zhang et al., 2020
*Triticum aestivum*	Drought/Cold	TabZIP60	A	Unknown	Positive regulation	Enhance drought and cold tolerance	Zhang L. N. et al., 2015
*Triticum aestivum*	Cold	TabZIP6	C	*CORs*	Negative regulation	Participate in cold stress response	Cai et al., 2018
*Triticum aestivum*	Cold	TabZIP14-B	C	Unknown	Positive regulation	Enhance cold tolerance	Zhang L. N. et al., 2017
*Triticum aestivum*	Cold/ Drought	TaAREB3/ TaABI5L2	A	*RD29A, RD29B, COR15A, COR47*	Positive regulation	Enhance cold and drought tolerance	Wang J. et al., 2016
*Triticum aestivum*	Cold	TaABL1 (ABI-like)	A	Unknown	Positive regulation	Enhance cold tolerance	Xu et al., 2014; Banerjee et al., 2017
*Tartary Buckwheat*	Salt/ Drought	FtbZIP5	A	*RD29A*, *RD29B, RAB18, RD26*, *RD20, COR15*	Positive regulation	Enhance salt and drought tolerance	Li et al., 2020
*Tartary Buckwheat*	Salt/ Drought	FtbZIP 83	A	*AtRD29A, AtRD29B, AtRD20, AtAIL, AtRAB18, AtKIN2, AtABI1, AtABI2*	Positive regulation	Enhance salt and drought tolerance	Li et al., 2019
*Vitis vinifera*	Osmotic stress	VvABF2	A	Unknown	Positive regulation	Enhance osmotic tolerance	Liu J. Y. et al., 2019
*Zea mays*	Salt/ Drought	ZmABP9	A	Unknown	Positive regulation	Enhance salt and drought tolerance	Zhang X. et al., 2011; Wang C. et al., 2017; Zong et al., 2020

## bZIP TFs Involved in Salt Stress Response

Under salt stress, plant cell should successively face challenges of osmotic stress, ion toxicity and oxidative stress (Munns, 2005; Rozema and Flowers, 2008). In these responses, bZIP transcription factors play key roles in various physiological processes in *Arabidopsis thaliana*, tomato, tobacco, rice, and soybeans, etc.

In *Arabidopsis thaliana*, AtbZIP17 was proven as a positive regulator in the processes salt stress responses, it activates both the expression of salt stress response gene *ATHB-7* and *SES1* (Liu et al., 2007; Liu et al., 2008); while the AtbZIP24 was revealed as a negative regulator in plant tolerance to salinity (Yang et al., 2009). Tang W. et al. (2012) found that heterologously expressing *Arabidopsis thaliana AtbZIP60* could increase salt resistance and superoxide dismutase activity of tobacco, rice, and *Pinus elliottii*. Recently, Rolly et al. (2020) found that AtbZIP62 negatively regulated the transcriptional SOS signaling pathway genes and thus negatively regulates the salt tolerance of Arabidopsis. In *Glycine max*, overexpression of the *GmbZIP1* enhances salt tolerance in transgenic plants (Gao et al., 2011). The overexpression of *GmbZIP2* in soybean hairy roots could enhance the expression of the stress responsive genes *GmMYB48, GmWD40*, *GmDHN15*, *GmGST1*, and *GmLEA*, thereby improving plant resistance to drought and salt stresses (Yang et al., 2020). Besides, heterologously expressing *GmbZIP44*, *GmbZIP62*, and *GmbZIP78* could significantly increase salt resistance of transgenic *Arabidopsis thaliana* plants (Wang et al., 2015). In maize, the *ABP9* was found as a salinity responsible bZIP gene by Zhang et al. (2011a). Then, Wang et al. (2017a) heterologously expressed it to improve the salt tolerance of transgenic cotton. In *Oryza sativa*, the *OsbZIP05/OSBZ8* firstly found with a higher transcriptional level in salt tolerant cultivar than in salt sensitive cultivar, indicate that *OsbZIP05/OSBZ8* might play as a positive role in this stress responses (Mukherjee et al., 2006). After that, OsbZIP12/OsABF1, OsbZIP23, OsbZIP46/OsABF2, OsBZIP71, and OsbZIP72 were successively proven to act as positive regulators in the process of salt tolerance (Xiang et al., 2008; Lu et al., 2009; Amir Hossain et al., 2010; Hossain et al., 2010; Tang N. et al., 2012; Liu C. T. et al., 2014; Chang et al., 2017; Zhang C. Y. et al., 2017). OsbZIP71 can form both homodimers and heterodimers with Group C members of the bZIP gene family, and overexpression of *OsbZIP71* can significantly enhance the salt tolerance of transgenic rice (Liu C. T. et al., 2014). On the contrary, the plants overexpressing *OsbZIP10/OsABI5* showed more obvious chlorosis than wild type under high salt concentration, indicating that *OsbZIP10/OsABI5* participates in the salt stress tolerance response of rice as a negative regulator (Zou et al., 2008).

Recent years, bZIPs in other plants have also been revealed to participate salinity responsive processes. Cheng et al. (2013) isolated a salt responsive transcriptional factor LrbZIP in lotus root and found that transgenic lotus with *LrbZIP* overexpression could grow with normal root biomass, chlorophyll content, and electrolyte exudation rate under NaCl treatment. Zhao et al. (2016) revealed that *Brassica napus* bZIP transcription factor BnaABF2 enhanced salt tolerance of plants through the ABA pathway. Gai et al. (2020) demonstrated that overexpression of the pepper *CabZIP25* enhanced the germination rate, fresh weight, chlorophyll content, and root lengths under salt stress.

To sum up, many bZIP genes have been excavated in different plants and confirmed that they can significantly enhance the salt tolerance of plants, making the bZIP gene family a gene treasure house for improving the salt tolerance of crops. Therefore, the use of bZIP transcription factors to improve the salt tolerance of crops and breed new salt-tolerant varieties is of great significance for improving agricultural productivity and improving saline soils.

## bZIP TFs Involved in Drought Stress Response

Drought is an adverse environmental factor that threatens plant growth and development. Many plant bZIP family members are involved in response to drought stress. Series of studies have shown that several rice bZIP transcription factors are involved in drought resistance. Liu J. Y. et al. (2014) found that rice *OsbZIP71* directly binds to the promoters of *OsNHX1* and *COR413-TM1* and activates their transcription so as to enhance drought resistance of transgenic rice. Yang et al. (2019a) showed that overexpression of *OsbZIP62* enhanced the drought tolerance and oxidative stress tolerance of transgenic rice. Except rice, some drought-related bZIP transcription factor genes cloned in other plants also significantly enhanced the drought resistance of transgenic crops. Overexpression of maize *ABP9* confers excellent drought tolerance to transgenic *Arabidopsis thaliana* plant (Wang C. et al., 2017). Under drought stress, the transgenic Arabidopsis plants of *IbbZIP1* showed significant upregulation of the genes involved in ABA and proline biosynthesis and reactive oxygen species scavenging system, so as to significantly decrease of H_2_O_2_ content (Kang et al., 2019). During seed germination and plant development, transgenic ramie plants overexpressing *BnbZIP2* were more sensitive to drought stress than wild-type (Huang et al., 2016). In addition, overexpression of transcription factors such as *Arabidopsis thaliana* ABF3 (Wang Z. et al., 2016) and wheat TabZIP60 (Zhang L. N. et al., 2015) in plants can significantly improve the drought resistance of transgenic plants. On the contrary, Lim et al. (2018) found that the pepper bZIP transcription factor CaDILZ1 plays a negative regulatory role in response to drought stress.

## bZIP TFs Involved in Cold Stress Response

Low temperature stimulation will disturb the normal physiological and metabolic activities and further affect the plant growth and development. The plant mainly responds to low temperature stress through the ICE-CBF-COR pathway. Low temperature induces CBFs (C-repeat-binding Factors) expression by ICE (inducer of CBF expression), which recognizes CRT/DRE (C-repeat/dehydration responsive *cis* element) located on the promoter of *COR* (cold regulated) genes (Shi et al., 2018). bZIP transcription factors also play indispensable roles in regulating plant cold stress responses.

The first rice bZIP-like transcription factor identified and reported was OsbZIP38/LIP19 of the H subfamily. As a Fos-like molecular switch, it is involved in the plant’s response to cold signal pathways (Aguan et al., 1991; Aguan et al., 1993; Shimizu et al., 2005). OsbZIP38/LIP19 and OsbZIP87/OsOBF1 are more likely to form heterodimers to participate in the plant’s response to cold signaling (Shimizu et al., 2005). In addition, the rice OsbZIP52/RISBZ5, OsbZIP68/ROS-bZIP1, and OsbZIP73/OsTFX1 were also involved in cold resistance. As a member in the G subfamily, OsbZIP52/RISBZ5 is not induced by drought, salt, PEG, and ABA, but by low temperature. It can form homodimers and specifically bind G-box. However, the survival rate of rice plants over-expressed *OsbZIP52/RISBZ5* were significantly lower than those of wild type, indicating that *OsbZIP52/RISBZ5* negatively regulates the rice cold tolerance (Liu et al., 2012). Cheng et al. (2007) found that OsbZIP68/ROS-bZIP1 could be induced and responded quickly within 24 h when rice was treated at 10°C. Liu et al. (2018, 2019a) identified eight low temperature resistant bZIP genes in rice, including *OsbZIP08*, *OsbZIP35*, *OsbZIP38*, *OsbZIP46*, *OsbZIP63*, *OsbZIP72*, *OsbZIP73*, and *OsbZIP76*.

Except for rice, carrot, soybean, wheat, tomato, and other crops have also been successively excavated bZIP transcription factors in response to low temperature stress. For example, Ito et al. (1999) found that the expression of bZIP-like protein Lip (Low temperature-Induced protein) in the roots of radish was up-regulated under low temperature treatment, thereby enhancing its cold resistance. Soybeans GmbZIP44, GmbZIP62 and GmbZIP78 can regulate and promote the synthesis of proline (plant cold tolerance osmotic regulator) to enhance the plant tolerance to cold stress by activating the expression of downstream genes *ERF5*, *KIN1*, *CORl5A*, and *COR78* (Liao et al., 2008). Hwang et al. (2014) treated *Brassica rapa* with low temperature stress and found that the expression of 27 *BrbZIP*s were significantly up-regulated, among which *Bra000256*, *Bra003320*, *Bra004689*, *Bra011648*, *Bra020735*, and *Bra023540* may be the key genes involved in the response to this stress. Compared with wild-type *Arabidopsis thaliana*, heterologous expression of *TabZIP6* in wheat under cold treatment significantly reduced the expression of *CBFs*, key *CORs*, and other genes in transgenic plants, making the transgenic plants sensitive to low temperature (Cai et al., 2018). However, the over-expressed wheat *TabZIP14-B*, *TaAREB3*, and *TabZIP60* in *Arabidopsis thaliana* can significantly enhance the ability of plants to resist cold stress. In addition, transgenic plants are more sensitive to ABA than wild type, indicating that *TabZIP14-B*, *TaAREB3*, and *TabZIP60* all enhance the cold resistance of plants through the ABA pathway (Zhang L. N. et al., 2015; Wang J. et al., 2016; Zhang L. N. et al., 2017). Xu et al. (2014) found that over-expression of wheat bZIP transcription factor TaABL 1 (ABI-like) elevated cold tolerance in wheat. Apple bZIP transcription factor MdHY5 can respond to low temperature stress at both the transcriptional and protein levels. Overexpression of *MdHY5* can significantly enhance cold stress resistance in apple callus and transgenic *Arabidopsis thaliana*. EMSA results indicate that MdHY5 can bind to G-Box on the *MdCBF1* promoter, thereby increasing its transcription level *COR* genes independent of CBF (An et al., 2017b). Wang et al. (2017b) found that transgenic *Arabidopsis thaliana* plants showed reduced survival, increased electrical conductivity, increased malondialdehyde content, and reduced soluble sugar content when overexpressed *Camellia sinensis CsbZIP6* in it. Transcriptome analysis found that the expression of low-temperature and drought-responsive genes in over-expressed plants was significantly lower than that of wild type, indicating that *CsbZIP6* plays a negative regulatory role in low-temperature stress response. Recently, Yao et al. (2020) also discovered that CsbZIP18 is a negative regulator of freezing tolerance *via* an ABA-dependent pathway.

## bZIP TFs Involved in Osmotic Stress Response

Salinity and drought usually induce secondary damages, such as osmotic stress. Hence, it’s not difficult to understand that plant bZIPs also act as significant roles in response to osmotic stress.

The rice OsbZIP71 transcription factor recognizes and combines with the promoter of the osmo-regulatory gene *OsNHX1*, and further transports excess Na^+^ and K^+^ in the cytoplasm to the vacuole, reducing salt concentration in the cytoplasm to improve rice salt tolerance (Liu C. T. et al., 2014). In *Arabidopsis thaliana*, the AtbZIP63 can regulate protein-protein interactions to regulate the activity of proline dehydrogenase I, thereby enhancing the ability of the plant to tolerate hypotonic stress (Veerabagu et al., 2014); the VIP1 (AtbZIP51) rapidly accumulates in the nucleus in response to hypotonic stress (Hwang et al., 2014; Tsugama et al., 2016). Actually, VIP1/AtbZIP51 and bZIP29 can form a heterodimer to enhance their binding to the hypotonic response element (AGCTGK) in the promoters of osmotic response genes *CYP707A1* and *CYP707A3* (Van Leene et al., 2016). Furthermore, Tsugama et al. (2019) found that the VIP1/AtbZIP51 was dephosphorylated by PP2A (protein phosphatase 2A), so as to suppress mechanical stress-induced root waving.

## bZIP TFs Involved in Regulating ABA Signaling Pathway

As a ‘emergency hormone’ in plants, ABA is an important signaling molecule in plants. When plants encounter abiotic stress such as salt, drought, or low temperature, they will activate both ABA-dependent and ABA-independent signaling pathways (Shinozaki and Yamaguchi-Shinozaki, 1996; Bray, 1997; Thomashow, 1998; Verslues and Zhu, 2005). Genes involved in the ABA-dependent pathway not only induce ABA biosynthesis, but also regulate the expression of genes containing ABA response element binding factors (AREBs) (Zhu, 2002; Shinozaki and Yamaguchi-Shinozaki, 2007). The bZIP transcription factor family can bind to ABRE elements (Choi et al., 2000; Uno et al., 2000). So far, bZIP transcription factors are proven to participate in ABA-dependent stress signaling in various plants, including *Arabidopsis thaliana*, rice, soybean, wheat (Casaretto and Ho, 2003; Fujita et al., 2005; Kobayashi et al., 2008; Lu et al., 2009).

The A subfamily bZIP transcription factor in *Arabidopsis thaliana* is a major regulator of ABA-dependent responses (Satoh et al., 2004). AtbZIP1 regulates ABA signal transduction by binding to the ABREs and alters the expressions of the ABA responsive genes to tolerate the cold stress (Sun et al., 2011). In rice, OsbZIP23 and OsbZIP46 can directly regulate the expression of multiple stress genes through the ABA pathway, thereby significantly improving drought- and salt-resistance of rice (Xiang et al., 2008; Tang N. et al., 2012; Dey et al., 2016; Zong et al., 2016). OsbZIP23/66/72 positively regulates ABA-responsive genes through interacting with OsMFT2and promotes seed germination (Song et al., 2020). In the transgenic plants over-expressing *OsbZIP42*, it showed a rapid rise of transcriptional expression of ABA responsive *LEA3* and *Rab16* and increased tolerance to drought stress (Joo H. et al., 2019). In soybeans, GmbZIP44, GmbZIP62, and GmbZIP78 can positively regulate the expression of *ABI1* and *ABI2* genes and further induce the expression of downstream genes such as *ERF5*, *KIN1*, *COR15A*, and *COR78* in response to ABA treatment (Liao et al., 2008). In maize, the transcription factor NCP1 can interact with the ABRE-binding bZIP transcription activator ABP9 and inhibit its activity, then negatively regulating ABA signal and weakening plant tolerance to multiple stresses (Zong et al., 2020).

Recent years, bZIPs are also found with increasing contributions in regulating ABA responses in other plants. Joo H. et al. (2019, 2020) showed that the stability of bZIP transcription factor CaAIBZ1 and CaATBZ1 could be modulated by a RING-type E3 ligase, CaASRF1, so as to positively modulates abscisic acid (ABA) signaling and ABA-mediated drought response in pepper. Liu et al. (2019c) found that overexpression of the ABA-depended grapevine *VvABF2* gene could enhance osmotic stress tolerance in *Arabidopsis thaliana* and thereby reduce the cell membrane damage. Wang et al. (2019) found that sweet potato *IbABF4* gene, encodes a bZIP transcription factor, overexpression in *Arabidopsis thaliana* and sweet potato could enhance their tolerance to multiple abiotic stresses through the ABA signaling pathway. Li et al. (2019, 2020) showed that the *tartary buckwheat* bZIP genes, *FtbZIP83*, *FtbZIP5* were both positive regulators involved in drought or salt stress *via* an ABA-dependent signaling pathway. In short, bZIP family members play important roles in the abscisic acid signaling pathway under various stresses. A large number of studies have shown that bZIP transcription factors affect ABA biosynthesis through the ABA-mediated signal transduction pathways and thus improve plant stress resistances.

## bZIP TFs Involved in Antioxidant System

Actually, the antioxidant system is an effective way for bZIP transcription factors to respond to abiotic stresses in plants (Miller et al., 2008; Choudhury et al., 2013). Superoxide dismutase (SOD), peroxidase (POD) and catalase (CAT) are three groups of key enzymes that removes active oxygen from plants. Overexpressing the bZIP gene in plants can increase the activity of peroxidase POD and SOD and increase the content of soluble sugars and proteins; it can also increase the elimination of active oxygen, promote the accumulation of soluble penetrants (Choudhury et al., 2013). For example, over expression of pepper *CAbZIP1* gene in *Arabidopsis thaliana* can eliminate the active oxygen by regulating the degradation enzyme POD and CAT, so as to improve the drought resistance and salt resistance of transgenic plants (Lee S. C. et al., 2006). Under stress conditions, POD and SOD activities of transgenic tobacco plants overexpressing *Tamarix hispida ThbZIP1* were significantly increased, accompanied by an increase in soluble protein and sugar content. Studies have shown that the *ThbZIP1* gene was significant upregulated under high-salt conditions, so as to improve plant salt tolerance by effectively removing reactive oxygen free radicals and accumulating soluble osmotic substances (Ji et al., 2013). Compared with wild-type plants, the transgenic tobacco with *OsHBP1b* under salt treatment enhanced the SOD activity, which further improved the stability of the vacuolar membrane and the K^+^/Na^+^ ratio, and had a stronger anti-oxidative damage function (Lakra et al., 2015). Further, Das et al. (2019) demonstrated that transgenic rice plants over-expressing *OsHBP1b* exhibit better survival and favorable osmotic parameters under salinity stress than the wild type counterparts. Overexpressing *Poncirus trifoliata* PtrABF in tobacco can stably promote the expression of nine stress-responsive genes in tobacco, and significantly induce the expression of three antioxidant enzyme genes under drought stress, which can be better removals of active oxygen free radicals and in turn enhances the resistance of transgenic plants to drought (Huang et al., 2010).

To reveal the relevance between bZIP subfamilies and stress types, the functional annotated bZIPs were also classified into 13 verified clades followed the approach used by Corrêa et al. (2008) (**Table 2** and **Figure 1**). There is yet not any functional report on bZIPs in subfamilies H, J, and L on abiotic stresses. Among the rest 10 subfamilies, there are 8, 7, 6, and 3 of which involved in salinity, drought, cold and osmotic stress, respectively. The bZIPs for regulating salinity tolerance are most frequently found in subgroups A, D, G, and S; while for modulating resistances to both drought and osmotic stress are most members in subgroup A; and for controlling cold responses are most those from subgroups A, C and S (**Table 2**).

## Regulation of bZIPs on Metabolism of Flavonoids Involved in Stress Responses

Recently, a plenty of flavonoids show significant contributions to plant tolerances to abiotic stresses (Yamasaki et al., 1997; Agati et al., 2012; Yan et al., 2014; Pi et al., 2016; Pi et al., 2018; Pi et al., 2019). Flavonoids are widely distributed in the plant kingdom and are abundant in flowers, fruits, and leaves of many plants (Du et al., 2010). Based on the different oxygen rings and conformations of the basic molecular structure, flavonoids are generally divided into six categories: flavone, flavonol, isoflavone, flavanone, flavanol, and anthocyanidin (Rice-Evans and Miller, 2010). The starting substrate for plant flavonoid biosynthesis is derived from coumaroyl-CoA of the phenylpropane metabolic pathway and malonyl-CoA from acetyl-coenzymes. Under the action of chalcone synthase (CHS), they first form chalcone (Aoki et al., 2000), and then the naringenin is formed by the catalytic action of chalcone isomerase (CHI) (McKhann and Hirsch, 1994). Under the catalysis of cytochrome P450 monooxygenase (CPM) and other enzymes, naringen can be used as a major intermediate metabolite to synthesize other flavonoids (Akashi et al., 1999; Liu et al., 2003; Falcone Ferreyra et al., 2012; Lam et al., 2014; Uchida et al., 2015).

More than 10,000 plant flavonoids have been discovered (Aoki et al., 2000; Jiang et al., 2010). They play very important roles in plant resistance to various stress (Yamasaki et al., 1997; Agati et al., 2012; Yan et al., 2014). They could remove free radicals under ultraviolet radiation (Li et al., 1993; Treutter, 2005), improve seed storage capacity and prolong life (Debeaujon et al., 2000), change petal color (Mola et al., 1998), interfere with the polar distribution of auxin (Buer and Muday, 2004), and affect the accumulation and composition of fatty acids (Lian et al., 2017).

Early studies on the mechanism of flavonoids involved in stress resistance mainly focused on their regulations on response to ultraviolet radiation (Tattini et al., 2006; Mellway et al., 2009). Later, flavonoids were found with strong antioxidant activity (Treutter, 2006; Agati et al., 2007; Pourcel et al., 2007; Hernández et al., 2009). Since various stresses can cause excessive peroxide to accumulate in plants, the significant role of flavonoids in plants’ stress resistance attracts increasing interests (Qiu et al., 2008; Fasano et al., 2014; Watkins et al., 2014; Rai et al., 2016). Tattini et al. (2004) reported that European privet flavonoids as antioxidants respond to strong light and drought stresses. Li et al. (2011) found a conserved *trans*-acting element (G-box, CACGTG) in the promoter region of the chalcone synthase family gene (*AtCHS*) in *Arabidopsis thaliana*, which regulates the accumulation of H_2_O_2_ by responding to cGMP signals (Abu Zahra et al., 2014). Yan et al. (2014) found that the cytochrome P450 monooxygenase GmFNSII/GmCPM in soybean was beneficial to the accumulation of flavonoid aglycones in plants and the reduction of H_2_O_2_ content. In previous studies, we found that the content of flavonoids such as quercimeritrin in salt-tolerant soybeans is relatively higher than that of salt-sensitive soybeans, which is beneficial for soybeans to adapt to salt stress (Lu et al., 2013). We further discovered that enzymes related to the flavonoid metabolism pathway are important salt stress response factors, and they can significantly regulate the salt tolerance of plants such as *Arabidopsis thaliana* and soybean (Pi et al., 2016). We recently found that the salt-triggered phosphorylation of GmMYB173, subsequent elevates the transcription of *GmCHS5* for enhancing the accumulation of dihydroxy B-ring flavonoids (such as cyaniding-3-arabinoside chloride) (Pi et al., 2018); while salt-inhibited phosphorylation of GmMYB183 subsequently decreases the transcription of *GmCYP81E11* for reducing monohydroxy B-ring flavonoids (such as ononin) (Pi et al., 2019). Actually, both GmMYB173 phosphorylation and GmMYB183 dephosphorylation contribute to soybean salt tolerance.

The abovementioned studies showed that flavonoids played very important roles in plant responses to stress. Interestingly, many bZIP transcription factors usually play key regulatory roles in the process of flavonoid biosynthesis. They regulate the expression of key enzyme genes in the synthetic pathway, thereby regulating the metabolism and synthesis of flavonoids.

Matousek et al. (2010) found that both hop HlbZIP1 and HlbZIP2 could activate the expression of chalcone synthase *chs_H1* and the *O-methyl transferase 1* genes and further regulate the accumulation of flavonoid glycosides and anthocyanins. Akagi et al. (2012) found that ectopic *DkbZIP5* overexpression in persimmon calluses could induced the up-regulation of *DkMyb4* and then affect the seasonal biosynthesis of proanthocyanidins in persimmon fruit. Malacarne et al. (2016) showed that VvibZIPC22, a member of clade C of the grapevine bZIP family, was able to activate the transcriptional expression of specific genes of the flavonoid pathway including *VviCHS3*, *VviCHI*, *VviFLS1*, and *VviANR*, alone or together with other factors to participate in the biosynthesis of flavonols during flowering and UV light-mediated induction. Dash et al. (2017) found that the poplar PatbZIP1 transcription factor regulated the expression of two flavonol synthase genes *PtaFLS2* and *PtaFLS4* and thus promotes the lateral root formation. bZIP transcription factor HY5 plays a multifaceted role in plant growth and development. Apple *MdHY5* gene, induced by light and abscisic acid treatments, promoted anthocyanin accumulation by regulating expression of the *MdMYB10* gene and downstream anthocyanin biosynthesis genes (An et al., 2017a). Zhang et al. (2011b) found that two bZIP transcription factors AtbZIP56/HY5 and AtbZIP64/HYH in *Arabidopsis thaliana* induced the accumulation of anthocyanins under low temperature. In addition, ABA can induce the expression of *Artemisia annua AabZIP1* to activate the expression of downstream gene *ADS* and *CYP71AV1*, thereby regulating the biosynthesis of artemisinin (Zhang F. Y. et al., 2015). Fan et al. (2019) showed that the expression of *RsbZIP011* and *RsbZIP102* were significantly up-regulated in radish tissue with higher anthocyanin content under heat and salt stress.

So far, the bZIPs that involve in flavonoid synthesis varies from plant species and their target genes (coding for different enzymes in flavonoid metabolism). To uncover the relationship between bZIP subfamilies and flavonoid synthesis, all the functional annotated bZIPs were also categorized into the 13 known subgroups according to Corrêa et al. (2008) (**Table 3** and **Figure 1**). It seems that only bZIPs in subfamilies A, H, and S might regulate flavonoid metabolism.

**Table 3:** Regulation of bZIP transcription factors on metabolism of flavonoids.

Species	Nomenclature	Subfamily	Target gene	Function	Reference
*Arabidopsis thaliana*	AtbZIP56/HY5, AtbZIP64/HYH	H	Unknown	Induce the accumulation of anthocyanins	Zhang Y. Q. et al., 2011
*Artemisia annua*	AabZIP1	A	*ADS, CYP71AV1*	Regulate the biosynthesis of artemisinin	Zhang F. Y. et al., 2015
*Diospyros kaki*	DkbZIP5	A	*DkMyb4*	Affect the seasonal biosynthesis of proanthocyanidins in persimmon fruit	Akagi et al., 2012
*Humulus lupulus*	HlbZIP1, HlbZIP2	A	*Chs_H1, O-methyl transferase 1*	Regulate the accumulation of flavonoid glycosides and anthocyanins	Matousek et al., 2010
*Malus pumila*	MdHY5	H	*MdMYB10*	Promote anthocyanin accumulation	An et al., 2017a
*Populus*	PatbZIP1	A	*PtaFLS2, PtaFLS4*	Promote the synthesis of related flavonoids and thus promotes the lateral root Formation and promotion of poplar biomass	Dash et al., 2017
*Raphanus sativns*	RsbZIP011, RsbZIP102	H	Unknown	Participant in the anthocyanin biosynthetic pathway	Fan et al., 2019
*Vitis vinifera*	VvibZIPC22	S	*VviCHS3, VviCHI, VviFLS1, VviANR*	Participate in the biosynthesis of flavonols	Malacarne et al., 2016

## Concluding Remarks

Due to their significant roles in plant tolerances to various stresses, the bZIP transcription factors have been comprehensively studied, including their categorization and regulatory mechanisms of target genes. However, there is at least one interesting issue worthy of further investigation: whether bZIP transcription factor regulates plant stress tolerance by modulating the synthesis of flavonoids.

To date, plenty of literatures show that bZIPs regulate plant tolerances to various abiotic stresses, such as low temperature, drought, high salt, nitrogen deficiency, zinc deficiency time (Lilay et al., 2020; Ueda et al., 2020). Besides, there are many reports reveal that flavonoids participate in various stress responses. Moreover, a lot of researches have now confirmed that bZIP transcription factors play an important role in the synthesis of flavonoids. Specially, bZIPs in subfamily H could bind to G-box in promoter of cold responsive genes (**Tables 1** and **2**); members of this subfamily also could modulate the synthesis of some flavonoids (**Table 3**). Since members in this group shares similar conversed protein motifs (**Supplemental Figures S1** and **S2**), it is reasonable to hypothesize that plant bZIPs in subfamily H could bind to G-box of cold-responsive genes to further regulate the synthesis of flavonoids. Similarly, it also makes sense that bZIPs in subfamily A could regulate the synthesis of flavonoids by binding to G-box or ABRE *cis*-elements of target genes involved in cold, salinity, drought and osmotic stresses; subfamily S could regulate the synthesis of flavonoids by bind to G-box or C-box or A-box or ABRE of genes involved in cold, salinity, and drought stresses (**Tables 1**–**3**). However, these hypotheses are still needed to be further verified.

## Author Contributions

YY completed the writing of this article. YQ, MJ, and JY assisted in the data collection and table making. JX, TZ, and LG took charge of the drawing. EP is responsible for the revision of this article.

## Funding

This work was supported by the grants 31970286 and 31301053 from the National Science Foundation of China, LY17C020004 from the Natural Science Foundation of Zhejiang Province, 20170432B01 from the Hangzhou Science and Technology Bureau, PF14002004014, PD11002002018001, 2016XJSGWXM27 and 2016XJSGWXM32 from Hangzhou Normal University.

## Conflict of Interest

The authors declare that the research was conducted in the absence of any commercial or financial relationships that could be construed as a potential conflict of interest.
